# Is the JAK-STAT Signaling Pathway Involved in the Pathogenesis of Depression?

**DOI:** 10.3390/jcm11072056

**Published:** 2022-04-06

**Authors:** Małgorzata Gałecka, Janusz Szemraj, Kuan-Pin Su, Angelos Halaris, Michael Maes, Aleksandra Skiba, Piotr Gałecki, Katarzyna Bliźniewska-Kowalska

**Affiliations:** 1 Department of Psychotherapy, Medical University of Lodz, 91-229 Lodz, Poland; malgorzata.galecka@umed.lodz.pl; 2 Department of Medical Biochemistry, Medical University of Lodz, 92-215 Lodz, Poland; janusz.szemraj@umed.lodz.pl; 3 College of Medicine, China Medical University, Taichung 404, Taiwan; cobol@cmu.edu.tw; 4 An-Nan Hospital, China Medical University, Tainan 709, Taiwan; 5 Department of Psychiatry and Behavioral Neuroscience, Stritch School of Medicine, Loyola University Chicago and Loyola University Medical Center, Maywood, IL 60153, USA; ahalaris@luc.edu; 6 Department of Psychiatry, Faculty of Medicine, Chulalongkorn University, Bangkok 10330, Thailand; dr.michaelmaes@hotmail.com; 7 Department of Adult Psychiatry, Medical University of Lodz, 91-229 Lodz, Poland; aleksandra.skiba@umed.lodz.pl (A.S.); piotr.galecki@umed.lodz.pl (P.G.)

**Keywords:** depression, JAK-STAT pathway, Janus kinase (JAK), signal transducer and activator of transcription (STAT) proteins, immunity

## Abstract

(1) Background: Only 60–70% of depressed patients respond to standard antidepressant treatments. Hence, it is essential to search for new, effective and safe therapies for unmet clinical needs of treatment-resistant depression (TRD). Agents targeting the components of the JAK-STAT signaling pathway have been shown to be relevant in immunology and are commonly used in the treatment of many hematological, rheumatological and dermatological diseases. The aim of this study was to investigate the role of elements of the JAK-STAT signaling pathway in the etiopathogenesis of depressive disorders. (2) Methods: A total of 290 subjects took part in the study (190 depressed patients, 100 healthy controls). Sociodemographic data were collected. The severity of depressive symptoms was assessed using the Hamilton Depression Rating Scale (HDRS). The gene expression at the mRNA protein levels of JAK (JAK1-JAK3) and STAT (STAT1-STAT5) was assessed by using RT-PCR and ELISA. (3) Results: Increased expression of JAK3 and decreased expression of STAT1 were observed in the group of depressed patients. (4) Conclusions: Further studies are necessary to determine whether moderation of the JAK-STAT signaling pathways is involved in the treatment of depression.

## 1. Introduction

Depressive disorders are the most common mental health conditions [1]. They constitute a serious medical and socioeconomic problem, as they lead to lower productivity and an increase in health care costs [2]. Therefore, effective treatment of depression is extremely important not only from the point of view of patients and their families, but the entire society. Research shows that only two-thirds of patients suffering from depressive disorders respond to standard antidepressant treatment, which means that treatment-resistant depression (TRD) may affect up to one-third of patients with depressive symptoms [3,4]. Hence, in order to develop new, effective and safe therapeutic agents and modalities, further research in the etiopathology of depression development and potential factors leading to drug resistance is very important. 

Dysregulation of the immune system is considered to be one of the most important factors contributing to treatment-resistant depression (TRD) [4]. The importance of the immune system in the etiopathology of depression, well documented in numerous studies [5,6], suggests that the introduction of biological therapies targeting elements of the immune system may be the missing link in the effective pharmacotherapy of depression, especially TRD.

The JAK-STAT signaling pathway plays a key role in processes such as immunity, cell division, cell death and cancer. It is a system of intracellular proteins used by many cytokines and growth factors to induce the expression of specific genes. The combination of a ligand with a membrane receptor activates the associated tyrosine kinase (JAK, Janus activated kinase, originally just another kinase), which activates the cytoplasmic domain of the receptor through phosphorylation. In turn, the STAT proteins bind to active domains, which then, in the dimerization process, detach from it and reach the cell nucleus to bind to the appropriate promoter and initiate the process of gene transcription. Hence, the expansion of the abbreviation STATs means signal transducer and activator of transcription proteins [7,8,9].

In humans, there are four members of the JAK family (JAK1, JAK2, JAK3 and TYK2-tyrosine kinase 2) and as many as seven STAT proteins: STAT1, STAT2, STAT3, STAT4, STAT5 (STAT5A and STAT5B) and STAT6. Various JAK and STAT proteins are recruited depending on the type of tissue and receptors involved in a given process [8,9].

The great importance of JAK kinases in immunological processes is evidenced by the fact that human JAK mutations cause numerous diseases, including severe combined immunodeficiency, Hyper IgE syndrome, some leukemias, polycythemia vera and other myeloproliferative disorders [10]. Due to their causal role in these diseases and their central role in the immune response, JAKs have become an attractive target for the development of therapies for various disorders of the hematopoietic and immune systems [11,12]. Anti-JAK monoclonal antibodies are also used in the treatment of autoimmune diseases of the joints and skin [13,14].

Depression is often associated with autoimmune diseases, such as psoriasis or rheumatoid arthritis (RA) [15]. Researchers are still seeking to confirm common pathological mechanisms of these disorders and common targeted therapeutic methods [5].

The aim of this study was to assess the importance of the JAK-STAT signaling pathway in the etiopathogenesis of depressive disorders by comparing the mRNA and protein expression of genes for individual elements of this pathway (JAK1-JAK3 and STAT1-STAT5) in depressed patients and healthy subjects.

## 2. Materials and Methods

### 2.1. Subjects and Data Collection

A total of 290 participants participated in the study. The study group included 190 patients (117 F, 73 M) with the diagnosis of a depressive episode or recurrent depression disorder (F32 and F33, respectively, according to ICD-10 criteria) [16]. The control group consisted of 100 healthy volunteers (66 F, 34 M) with no depressive symptoms and a negative history for mental disorders. No statistical differences were observed between genders in both groups (*p* = 0.4583). Table 1 shows statistical characteristics of both study groups. The exclusion criteria were as follows: other psychiatric diagnoses than depressive disorders, serious neurological or somatic diseases that could affect the expression of selected genes, abuse of or addiction to psychoactive substances. 

Participants were native Poles (not related). They were chosen for the study group at random. Participation in the study was voluntary. Written informed consent for participation was obtained from each subject according to the study protocol approved by the Bioethical Committee of the Medical University of Lodz (No. RNN/833/11/KB).

### 2.2. Hamilton Depression Rating Scale (HDRS)

The mental state of all participants was evaluated on the day of inclusion in the study by a qualified psychiatrist. The severity of depressive symptoms in patients from the study group was assessed using the 17-item Hamilton Depression Rating Scale [17]. Participation in the study was not associated with any change in the antidepressant therapy.

### 2.3. Biochemical Measurements 

Peripheral venous blood samples were taken from all participants. RT-PCR was used to assess gene expression at the mRNA level, while ELISA was used to assess expression at the protein level. The obtained results were analyzed statistically in order to determine the correlation between gene expression and clinical and sociodemographic variables. 

#### 2.3.1. mRNA Expression Determination

InviTrap Spin Universal RNA Kit (Stratec molecular, Berlin, Germany) was used to isolate total ribonucleic acid (RNA) from the participants’ blood, and the procedure was performed according to the producer’s manual. The quantity and purity of isolated RNA was estimated spectrophotometrically (Picodrop-VWR International Corporate LLC, Radnor, PA, USA). The quality of samples was assessed using Agilent RNA 6000 Nano Kit on 2100 Bioanalyzer (Agilent Technologies–Santa Clara, CA, USA) in accordance with the manufacturer’s recommendations. Electrophoretogram and RIN values were used to determine the level of degradation of total RNA. Solely the samples with RIN value > 7 were subject to further analysis. Isolated RNA was stored at −70 °C.

TaqMan^®^ RNA Reverse Transcription Kit (Applied Biosystems, Foster City, CA, USA) was used to reverse transcribe the RNA samples into complementary DNA (cDNA) according to the manual supplied by the manufacturer. The reaction was performed using a Biometra TA advanced thermocycler (Analytik Jena, Jena, Germany), and the obtained cDNA was stored at −20 °C.

Real-time polymerase chain reaction (PCR) was managed using TaqMan^®^ Universal PCR Master Mix, No UNG (Applied Biosystems, Foster City, CA, USA) based on the manufacturer’s recommendations. Specific probes, i.e., Hs01026983_m1, Hs01078136_m1, Hs01006621_g1, Hs01013936_m1, Hs01013116_g1, Hs00374280_m1, Hs01028011_m1, Hs00560026_m1, Hs04194366_g1 for human JAK1, JAK2, JAK3, STAT1, STAT2, STAT3, STAT4, STAT5 and RPL13A genes, respectively, were delivered by Applied Biosystems. Reactions were performed on ABI 7000 Real-Time PCR (Applied Biosystems) and analyzed using ABI Prism 7000 (SDS Software). Relative gene expression on mRNA level was calculated using delta Ct method and RPL13A as a reference gene [18]. Control samples without RT and with no template cDNA were performed with each assay [18,19]. Specificity of transcripts amplification was further confirmed by melting curve profiles.

#### 2.3.2. Protein Expression Determination

To assess the total protein concentration in blood plasma, Micro BCA Protein Assay Kit (Thermo Fischer Scientific, Waltham, MA, USA) was used. According to the manufacturer’s manual, 150 µL of the reaction mixture was added to tubes containing the same volume (150 µL) of serum. Previously, the serum sample was diluted 10 times in PBS and incubated for 2 h at 37 °C. The serum concentration of JAK1, JAK2, JAK3, STAT1,STAT2, STAT3, STAT4, STAT5 proteins was determined using Human JAK1 ELISA Kit (Novus Biologicals LCC, Centennial, CO, USA), Human JAK2 ELISA Kit (ThermoFischer Scientific), Human JAK3 ELISA Kit (MyBiosource, San Diego, CA, USA), Human STAT1,STAT3 ELISA Kit (Abcam Cambridge, UK), Human STAT2, Human STAT4 (LifeSpan Bioscences, Seattle, WA, USA) and Human STAT5 (ThermoFischer Scientific, Waltham, MA, USA) according to the protocols provided by the manufacturer. Protein concentration was estimated using an analytical curve for serum albumin. Both the examined samples and the control samples were measured in triplicate using Multiskan Ascent Microplate Photometer (Thermo Labsystems, Philadelphia, PA, USA) at λ = 562 nm, and total protein concentration was calculated from the standard curve equation. Additionally, β-actin protein concentration in the samples was determined with the aid of Human Actin Beta (ACTb) ELISA Kit (BMASSAY, Beijing, China), as stated in the manufacturer’s manual. The absorbance of the samples was measured using Multiskan Ascent Microplate Photometer (Thermo Labsystems) at λ = 450 nm. β-actin was utilized for endogenous control of protein concentration.

#### 2.3.3. Statistical Analysis

A chi-square test was carried out for contingency tables (groups vs. gender). Generalized linear models with robust standard errors were used to test differences in numerical traits between the studied groups. All the fitted models were controlled for age and gender. All empirical data, considering the gene expression, were log transformed before testing the hypothesis. A level of *p* < 0.05 was deemed statistically significant. Spearman’s rank correlation coefficient for integer numerical data and the Pearson correlation coefficient for continuous numerical variables were used. All the statistical procedures were performed by using Stata/Standard Edition, release 14.2 (StataCorp LLC, College Station, TX, USA).

## 3. Results

### 3.1. Mean mRNA Gene Expression 

**Figure 1:**
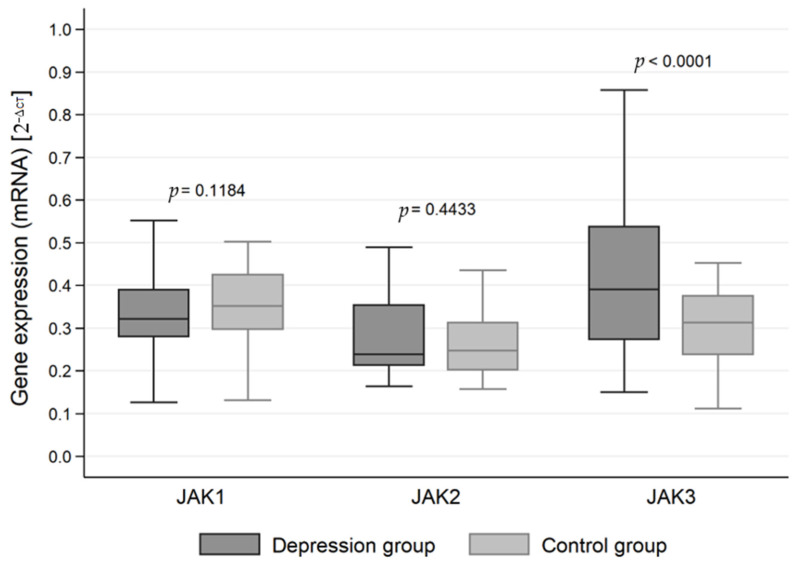
Expression of Janus kinase (JAK) genes at the mRNA level in the depression and healthy control group.

**Figure 2:**
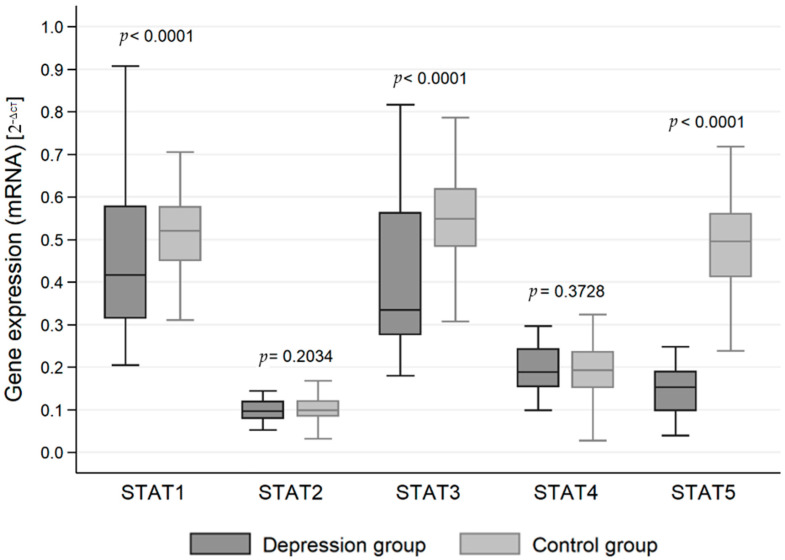
Expression of signal transducer and activator of transcription (STAT) protein family genes at the mRNA level in the depression and healthy control group.

The expression of genes at the mRNA level in the studied groups differed statistically significantly in the case of JAK3 (*p* < 0.0001), STAT1 (*p* < 0.0001), STAT3 (*p* < 0.0001) and STAT5 (*p* < 0.0001). JAK3 gene expression at the RNA level was statistically significantly higher in the study group than in the control group (0.413 vs. 0.306). On the other hand, the expression at the mRNA level of genes for the signal transducer and activator of transcription (STAT) proteins STAT1, STAT3 and STAT5 was statistically significantly higher in the control group than in the study group (0.509 vs. 0.444, 0.548 vs. 0.413 and 0.489 vs. 0.154, respectively) (Table 2, Figure 1 and Figure 2).

### 3.2. Mean Protein Expression 

**Figure 3:**
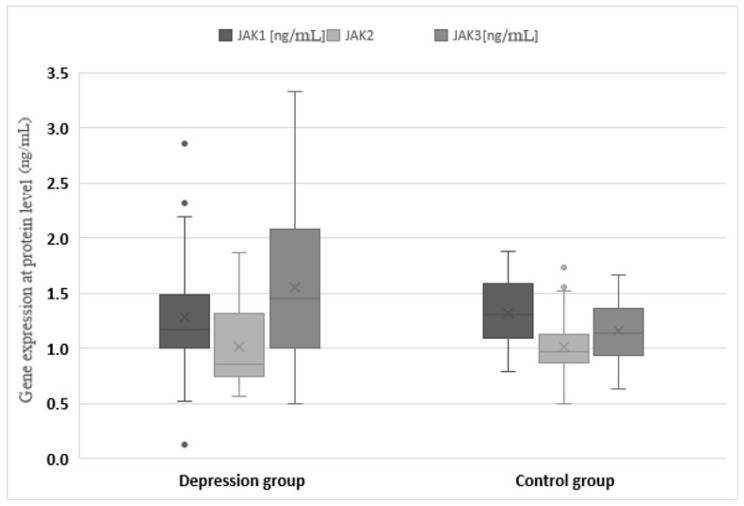
Expression of Janus kinase (JAK) genes at the protein level in the depression and healthy control group. Statistical significance of differences in study groups: JAK1 *p* = 0.2401; JAK2 *p* = 0.9553; JAK3 *p* < 0.0001.

**Figure 4:**
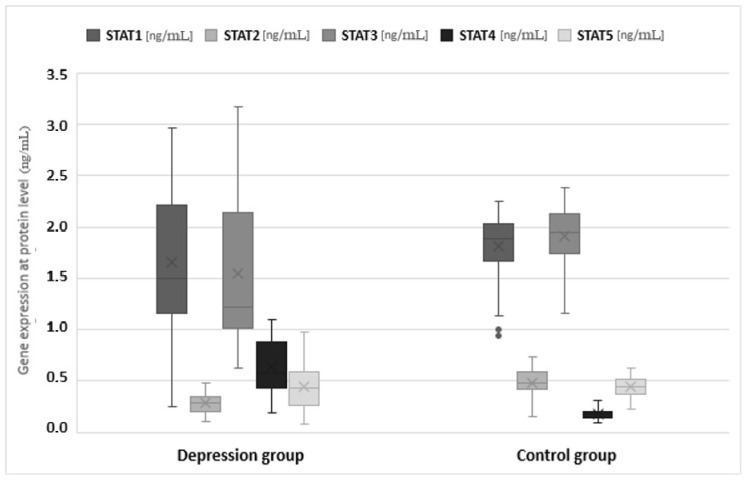
Expression of signal transducer and activator of transcription (STAT) protein family genes at the mRNA level in the depression and healthy control group. Statistical significance of differences in study groups: STAT1 *p* = 0.0120; STAT2 *p* < 0.0001; STAT3 *p* < 0.0001; STAT4 *p* < 0.0001; STAT5 *p* = 0.6931.

The expression of genes at the protein level in the studied groups differed statistically significantly in the case of JAK3 (*p* < 0.0001), STAT1 (*p* = 0.0120), STAT2 (*p* < 0.0001), STAT3 (*p* < 0.0001) and STAT4 (*p* < 0.001). JAK3 and STAT4 gene expression at the protein level was statistically significantly higher in the study group than in the control group (1.547 vs. 1.156 and 0.640 vs. 0.175, respectively). In turn, protein expression for STAT1, STAT2 and STAT3 was higher in the control group (1.811 vs. 1.652, 0.486 vs. 0.283 and 1.910 vs. 1.543, respectively) (Table 3, Figure 3 and Figure 4).

### 3.3. Correlation with Demographic and Clinical Variables

Gene expression and demographic (metric) data and clinical information. In the univariate and multivariate model, there was no statistically significant relationship between the expression of JAK1, JAK2, JAK3, STAT1, STAT2, STAT3, STAT4 and STAT5 genes in the above-mentioned independent variables in both groups (Table 4 and Table 5).

## 4. Discussion

JAK-STAT is a system related to the regulation of gene expression. Mainly identified in hematopoietic cells, however, its importance and role has also been recognized in all cell types, including neurons [20]. The pathway involves activation of the receptor by polypeptides, such as hormones, growth factors or cytokines that lead to JAK activation. JAK phosphorylates STATs, which then dimerize. The dimers travel to the nucleus where they bind to DNA and regulate transcription. This pathway involves various mechanisms, including the regulation of the phosphorylation state of JAK and STAT by phosphatases or the activity of JAK kinase by, inter alia, suppressor of cytokine signaling (SOCS) [20]. In the central nervous system (CNS), the JAK-STAT signaling pathway is mainly related to gene regulation during development. STAT expression in the CNS is weaker than in other systems, but various studies have shown that these proteins may be significant in several areas of the brain, including the cerebral cortex, hippocampus, hypothalamus and cerebellum. The expression of these proteins also changes during development. They are highly expressed at the embryonic stages (especially JAK2, JAK1, STAT3, STAT6 and STAT1), and their expression gradually decreases during growth and adulthood [21,22,23]. Recently, a protein–protein interaction network analysis showed that the activation of JAK-STAT signaling is an important pathway underpinning the immune disorders in major depression [24].

The factors released during inflammation are activators of the JAK-STAT signaling pathway. Oxidative stress and some cytokines (e.g., IL-6) activate both STAT1 and STAT3 through a JAK2-dependent mechanism [25]. The activation of this pathway is expected to promote the expression of genes related to inflammation, but it also regulates survival-related gene expression. Depending on the STAT isoform, this activation may have a different effect on inflammation, survival, proliferation or differentiation of cells. The role of STAT3 in encephalitis is still controversial. Research data indicate that they can either promote cell death and contribute to brain damage or be involved in the survival of neurons. The role of STAT1 is more consistent because it promotes cell death [26].

The importance and participation of signaling pathways in the pathogenesis of depression is confirmed by the fact that JAK can regulate the expression or function of several neurotransmitter receptors, including gamma-aminobutyric acid (GABA) [27], cholinergic muscarinic [28], N-methyl-D-aspartate (NMDA) and α-amino-3-hydroxy-5-methyl-4-isoxazolpropionic acid (AMPA) receptors, which are strongly associated with depressive symptoms [29,30,31].

Recent reports on major depression have led to the hypothesis that oxidative stress (OS) and inflammatory processes are involved in its development and may contribute to the dysfunction of the serotonergic and noradrenergic systems [32,33]. Oxidative stress has been found to play a key role in the etiopathology of various disease states and may be a common mechanism underlying several serious mental disorders [34]. The brain is more susceptible to the damaging effects of reactive oxygen species (ROS) due to its high metabolism, speed and low antioxidant levels, which may explain why oxidative stress is a major feature of most neurodegenerative diseases, including major depression [5,33].

Overproduction of ROS generates an inflammatory response and increases the release of pro-inflammatory cytokines. Furthermore, a large body of evidence has shown that major depression is characterized by disrupted inflammatory pathways, including cytokine expression, such as interleukin-1b (IL-1b), IL-6, interferon g (IFN-g) and tumor necrosis factor-a (TNF-a) or IL-17 [35,36]. Dysregulation of the JAK/STAT pathway has been found to be a key factor in various neurodegenerative diseases, which underlines its importance and determines how this pathway affects the fate and function of brain cells [20]. The JAK/STAT signaling pathway acts downstream of the binding of various ligands and cytokines growth factors, and ROS are inhibited by the action of the cytokine signaling suppressor (SOCS) [20].

Scientific research has confirmed that major depression has an inflammatory component, but due to the variety of possible inflammatory factors and the limited number of comprehensive studies, it is difficult to clearly define the importance of signaling pathways in development and occurrence of depression. Most studies that focus on immune diseases, such as rheumatoid arthritis, psoriasis or multiple sclerosis, and the key factors related to pathogenic differentiation of Th17 and Th22 cells and the production of IL-17 and IL-22 in their course, emphasize the pro-inflammatory importance of IL-6. -21 and -23 and STAT3, also due to the possibility of targeting these mediators in biological therapies [37]. Our research has confirmed that IL-17 is important in the course and development of depression, especially drug-resistant and recurrent depression. It was significantly higher in patients diagnosed with depression, and its increase was strongly associated with the duration of the disease and the number of hospitalizations [5]. Earlier studies also confirmed a strong relationship between depression and an increase in Il-6 and TNF-α, but their increase, according to researchers, was similar regardless of whether it was the first depressive episode or a subsequent one [38]. This motivates our interest in the therapeutic target of signaling pathways to treat depression, which may be a new area in the search of therapeutic pathways of recurrent and drug-resistant depression. Among the relationships we observed, the statistically significantly higher expression of Janus kinase 3 (JAK3) in patients with depressive disorders is noteworthy. This tyrosine kinase mediates important signaling events in both innate and adaptive immunity and plays a key role in the activation of T lymphocytes, which is characteristic of autoimmune diseases. It is involved in signaling by association with receptors for interleukins, such as IL2, IL4, IL7, IL9, IL15 and IL21.

The JAK-STAT pathway is strongly related to cell proliferation and differentiation. It is important for their survival, as well as for the response in case of inflammation [39]. It is also one of the most important factors involved in the regulation of the functioning of neurons [20]. Its dysregulation in the case of changes in the brain was clearly observed in both animal and human models, emphasizing its great therapeutic potential. However, further extensive research is needed to better understand how the JAK-STAT pathway affects the brain.

## 5. Conclusions

At both the mRNA and protein levels, increased expression of the gene for Janus kinase 3 (JAK3) and decreased expression of the gene for STAT1 were observed in the group of depressed patients compared to the control group of healthy subjects. Due to the importance of the JAK/STAT pathway in inflammatory diseases and the obtained results, this area is a new interesting therapeutic target in depression.

## Figures and Tables

**Table 1:** Demographic characteristics of the study cohort by nosology group.

Analyzed Trait	Study Group	Statistical Parameter *						
		M	Me	Q_1_–Q_3_ (IQR)	SD	SE	95% CI	Min.–Max.
Age (years) †	Test group	47.51	51	41–55 (14)	11.18	0.81	45.90–49.10	18–67
	Control group	29.36	26	24–30 (6)	8.71	0.87	27.63–31.10	20–53
	Overall	41.29	44	26–54 (28)	13.50	0.79	39.73–42.85	18–67
Number of hospitalizations	Test group only	2.01	1	1–2 (1)	2.00	0.15	1.72–2.30	0–12
Disease duration time (years)	Test group only	6.18	4	1–8 (7)	7.05	0.52	5.16–7.20	1–40
Number of episodes	Test group only	4.56	2	1–5 (4)	5.33	0.39	3.79–5.34	1–20
Hamilton Depression Rating Scale (HDRS)	Test group only	22.82	23	18–27 (9)	6.86	0.51	21.82–23.82	1–51

* Explanations of abbreviations used in result tables: M—mean; Me—median; Q—quartile; SD—standard deviation; SE—standard error; CI—confidence interval. † Statistical significance of differences: *p* < 0.0001 for the multifactor generalized linear model fitted, *p* < 0.001 for “by-group” comparison, *p* = 0.528 for “by-gender” comparison.

**Table 2:** Detailed descriptive statistics for mRNA gene expression (2^−∆CT^) by study group.

Gene	Study Group	Statistical Parameter *							
		M	Trim. M	Me	Q_1_–Q_3_ (IQR)	SD	SE	95% CI	Min.–Max.
JAK1	Test group	0.349	0.348	0.321	0.279–0.389 (0.110)	0.107	0.008	0.334–0.365	0.058–0.738
	Control group	0.354	0.354	0.351	0.296–0.425 (0.129)	0.090	0.009	0.336–0.372	0.131–0.628
	Overall	0.351	0.350	0.329	0.282–0.422 (0.140)	0.102	0.006	0.339–0.363	0.058–0.738
JAK2	Test group	0.281	0.278	0.238	0.21–0.354 (0.142)	0.093	0.007	0.268–0.295	0.163–0.707
	Control group	0.262	0.260	0.247	0.201–0.313 (0.112)	0.070	0.007	0.248–0.276	0.157–0.435
	Overall	0.274	0.271	0.238	0.211–0.336 (0.125)	0.086	0.005	0.264–0.284	0.157–0.707
JAK3	Test group	**0.413**	**0.409**	**0.391**	**0.273–0.538 (0.265)**	**0.164**	**0.012**	**0.389–0.436**	**0.149–0.858**
	Control group	**0.306**	**0.307**	**0.312**	**0.236–0.376 (0.140)**	**0.085**	**0.008**	**0.289–0.323**	**0.112–0.452**
	Overall	**0.376**	**0.371**	**0.333**	**0.261–0.488 (0.227)**	**0.150**	**0.009**	**0.358–0.393**	**0.112–0.858**
STAT1	Test group	**0.444**	**0.441**	**0.416**	**0.315–0.578 (0.263)**	**0.160**	**0.012**	**0.421–0.467**	**0.205–0.907**
	Control group	**0.509**	**0.511**	**0.520**	**0.450–0.577 (0.127)**	**0.100**	**0.010**	**0.489–0.528**	**0.233–0.705**
	Overall	**0.466**	**0.464**	**0.476**	**0.331–0.578 (0.247)**	**0.145**	**0.008**	**0.449–0.483**	**0.205–0.907**
STAT2	Test group	0.101	0.098	0.096	0.079–0.119 (0.040)	0.049	0.003	0.094–0.108	0.052–0.685
	Control group	0.116	0.103	0.099	0.084–0.120 (0.036)	0.110	0.011	0.094–0.137	0.010–0.915
	Overall	0.106	0.099	0.096	0.081–0.120 (0.039)	0.076	0.004	0.097–0.115	0.010–0.915
STAT3	Test group	**0.413**	**0.410**	**0.334**	**0.276–0.562 (0.286)**	**0.175**	**0.013**	**0.388–0.438**	**0.180–0.817**
	Control group	**0.548**	**0.549**	**0.548**	**0.483–0.619 (0.136)**	**0.100**	**0.010**	**0.529–0.569**	**0.307–0.786**
	Overall	**0.460**	**0.459**	**0.473**	**0.293–0.587 (0.294)**	**0.166**	**0.010**	**0.441–0.479**	**0.180–0.817**
STAT4	Test group	0.196	0.196	0.188	0.154–0.242 (0.088)	0.052	0.004	0.188–0.203	0.099–0.296
	Control group	0.195	0.196	0.192	0.152–0.236 (0.084)	0.063	0.006	0.182–0.207	0.019–0.324
	Overall	0.196	0.196	0.189	0.153–0.242 (0.089)	0.056	0.003	0.189–0.202	0.019–0.324
STAT5	Test group	**0.154**	**0.147**	**0.153**	**0.098–0.189 (0.091)**	**0.096**	**0.007**	**0.140–0.168**	**0.039–0.948**
	Control group	**0.489**	**0.490**	**0.495**	**0.412–0.561 (0.149)**	**0.110**	**0.011**	**0.467–0.511**	**0.238–0.718**
	Overall	**0.269**	**0.263**	**0.191**	**0.135–0.437 (0.302)**	**0.189**	**0.011**	**0.248–0.291**	**0.039–0.948**

* Explanations of abbreviations used in result tables: M—mean; Trim. M—trimmed mean; Me—median; Q—quartile; Q1—first quartile; Q3—third quartile; IQR—interquartile range; SD—standard deviation; SE—standard error; CI—confidence interval; Min—minimum; Max—maximum. Statistical significance of differences by study groups: JAK1 *p* = 0.1184; JAK2 *p* = 0.4433; JAK3 *p* < 0.0001; STAT1 *p* < 0.0001; STAT2 *p* = 0.2034; STAT3 *p* < 0.0001; STAT4 *p* = 0.3728; STAT5 *p* < 0.0001. Values in bold indicate significant difference. All empirical data, considering the gene expression, were log transformed before testing the hypothesis. All the models fitted were controlled for age and gender. Genes: JAK—Janus kina 1–3 s; STAT—signal transducer and activator of transcription protein 1–5.

**Table 3:** Detailed descriptive statistics for gene expression at the protein level (ng/mL) by study group.

Gene	Study Group	Statistical Parameter						
		M	Me	Q_1_–Q_3_ (IQR)	SD	SE	95% CI	Min.–Max.
JAK1	Test group	1.286	1.175	1.006–1.476 (0.470)	0.447	0.032	1.222–1.350	0.127–2.861
	Control group	1.321	1.307	1.093–1.582 (0.489)	0.288	0.029	1.264–1.378	0.783–1.883
	Overall	1.298	1.199	1.071–1.537 (0.466)	0.399	0.023	1.252–1.344	0.127–2.861
JAK2	Test group	1.017	0.853	0.742–1.313 (0.571)	0.353	0.026	0.966–1.068	0.558–1.867
	Control group	1.015	0.971	0.870–1.129 (0.259)	0.219	0.022	0.972–1.058	0.498–1.729
	Overall	1.016	0.898	0.773–1.221 (0.448)	0.313	0.018	0.981–1.051	0.498–1.867
JAK3	Test group	**1.547**	**1.448**	**0.997–2.079 (1.082)**	**0.646**	**0.047**	**1.455–1.640**	**0.499–3.329**
	Control group	**1.156**	**1.140**	**0.941–1.358 (0.417)**	**0.273**	**0.027**	**1.102–1.210**	**0.635–1.668**
	Overall	**1.412**	**1.227**	**0.979–1.807 (0.828)**	**0.577**	**0.034**	**1.346–1.479**	**0.499–3.329**
STAT1	Test group	**1.652**	**1.501**	**1.166–2.209 (1.043)**	**0.631**	**0.046**	**1.561–1.742**	**0.251–2.958**
	Control group	**1.811**	**1.883**	**1.667–2.023 (0.356)**	**0.299**	**0.030**	**1.752–1.870**	**0.938–2.249**
	Overall	**1.706**	**1.762**	**1.212–2.132 (0.920)**	**0.545**	**0.032**	**1.644–1.769**	**0.251–2.958**
STAT2	Test group	**0.283**	**0.289**	**0.205–0.344 (0.139)**	**0.086**	**0.006**	**0.271–0295**	**0.109–0.479**
	Control group	**0.486**	**0.480**	**0.415–0.593 (0.178)**	**0.120**	**0.012**	**0.642–0.509**	**0.158–0.735**
	Overall	**0.353**	**0.336**	**0.235–0.441 (0.206)**	**0.138**	**0.008**	**0.337–0.369**	**0.109–0.735**
STAT3	Test group	**1.543**	**1.220**	**1.010–2.140 (1.130)**	**0.696**	**0.050**	**1.444–1.643**	**0.630–3.170**
	Control group	**1.910**	**1.943**	**1.738–2.121 (0.383)**	**0.274**	**0.027**	**1.857–1.963**	**1.158–2.383**
	Overall	**1.670**	**1.726**	**1.085–2.129 (1.044)**	**0.611**	**0.036**	**1.599–1.741**	**0.630–3.170**
STAT4	Test group	**0.640**	**0.574**	**0.437–0.884 (0.447)**	**0.250**	**0.018**	**0.605–0.676**	**0.192–1.095**
	Control group	**0.175**	**0.172**	**0.140–0.208 (0.068)**	**0.048**	**0.005**	**0.165–0.184**	**0.098–0.307**
	Overall	**0.480**	**0.432**	**0.198–0.752 (0.554)**	**0.301**	**0.018**	**0.445–0.515**	**0.098–1.095**
STAT5	Test group	0.450	0.436	0.262–0.592 (0.330)	0.219	0.016	0.419–0.481	0.083–0.978
	Control group	0.443	0.439	0.374–0.522 (0.148)	0.089	0.009	0.425–0.461	0.231–0.628
	Overall	0.448	0.437	0.326–0.544 (0.218)	0.185	0.011	0.426–0.470	0.083–0.978

(Statistical significance of differences by study groups: JAK1 *p* = 0.2401; JAK2 *p* = 0.9553; JAK3 *p* < 0.0001; STAT1 *p* = 0.0120; STAT2 *p* < 0.0001; STAT3 *p* < 0.0001; STAT4 *p* < 0.0001; STAT5 *p* = 0.6931. Values in bold indicate significant difference. All the models carried out were controlled for age and gender.). Genes: JAK—Janus kina 1–3 s; STAT—signal transducer and activator of transcription protein 1–5.

**Table 4:** Spearman’s rank correlation coefficients, their confidence limits and levels of statistical significance for the mRNA gene expression vs. selected clinical data in the test group.

Test Group	mRNA Gene Expression							
Variables	JAK1	JAK2	JAK3	STAT1	STAT2	STAT3	STAT4	STAT5
Number of hospitalizations	0.040 (−0.105)–0.183 0.5897	0.039 (−0.106)–0.181 0.6012	−0.015 (−0.159)–0.129 0.8388	−0.106 (−0.247)–0.038 0.1479	−0.100 (−0.240)–0.045 0.1745	0.010 (−0.134)–0.154 0.8876	0.043 (−0.101)–0.186 0.5587	0.069 (−0.079)–0.211 0.3469
Disease duration time (years)	0.054 (−0.091)–0.196 0.4682	0.049 (−0.096)–0.191 0.5093	−0.029 (−0.172)–0.115 0.6938	−0.009 (−0.153)–0.135 0.9003	0.064 (−0.081)–0.206 0.3866	0.021 (−0.123)–0.164 0.7749	0.074 (−0.071)–0.216 0.3160	0.051 (−0.094)–0.193 0.4924
Number of episodes	0.015 (−0.129)–0.159 0.8339	0.118 (−0.026)–0.258 0.1075	0.005 (−0.139)–0.148 0.9512	−0.104 (−0.245)–0.040 0.1558	−0.095 (−0.236)–0.049 0.1954	0.125 (−0.019)–0.264 0.0891	0.082 (−0.063)–0.223 0.2675	0.047 (−0.098)–0.189 0.5271
Hamilton Depression Rating Scale (HDRS)	−0.102 (−0.243)–0.043 0.1678	0.057 (−0.089)–0.200 0.4439	−0.042 (−0.186)–0.103 0.5678	0.067 (−0.079)–0.210 0.3671	−0.022 (−0.166)–0.123 0.7686	0.076 (−0.069)–0.219 0.3036	−0.111 (−0.252)–0.034 0.1322	−0.026 (−0.170)–0.120 0.7303

**Table 5:** Pearson product–moment correlation coefficients, their confidence limits and levels of statistical significance for the mRNA gene expression vs. patients’ age in study group.

Correlation with Age (Years)	mRNA Gene Expression							
	JAK1	JAK2	JAK3	STAT1	STAT2	STAT3	STAT4	STAT5
Test group	−0.056 (−0.197)–0.087 = 0.4438	−0.019 (−0.161)–0.123 = 0.7909	−0.047 (−0.188)–0.096 = 0.5194	−0.041 (−0.182)–0.102 = 0.5752	−0.047 (−0.188)–0.096 = 0.5229	0.001 (−0.142)–0.143 = 0.9932	−0.012 (−0.154)–0.131 = 0.8737	−0.055 (−0.196)–0.088 = 0.4503
Control group	−0.211 (−0.392)–(−0.014) = 0.0360	−0.114 (−0.305)–0.085 = 0.2605	0.007 (−0.190)–0.204 = 0.9434	−0.094 (−0.286)–0.105 = 0.3529	−0.053 (−0.248)–0.146 = 0.6008	0.165 (−0.034)–0.351 = 0.1035	0.076 (−0.123)–0.269 = 0.4551	0.075 (−0.124)–0.268 = 0.4603
Overall	−0.096 (−0.164)–0.066 = 0.4037	−0.049 (−0.096)–0.019 = 0.1018	−0.230 (−0.336)–(−0.118) = 0.0001	0.124 0.008–0.236 = 0.0356	−0.006 (−0.122)–0.109 = 0.9148	0.296 0.187–0.398 < 0.0001	−0.023 (−0.138)–0.093 = 0.6982	0.512 0.422–0.593 < 0.0001

## Data Availability

The data analyzed in the study are available upon request from the authors of the article.
